# Disinclusion of unerupted teeth by mean of self-ligating brackets: Effect of blood contamination on shear bond strength

**DOI:** 10.4317/medoral.18246

**Published:** 2012-12-10

**Authors:** Andrea Scribante, Maria F. Sfondrini, Sara Gatti, Paola Gandini

**Affiliations:** 1 DDS, PhD. Department of Orthodontics and Department of Surgical Sciences (Head: Prof. Paolo Dionigi), University of Pavia, Pavia, Italy; 2 MD, DDS, PhD. Department of Orthodontics, University of Pavia, Pavia, Italy; 3 DDS. Department of Orthodontics, University of Pavia, Pavia, Italy; 4 MD, DDS, Head Professor, Department of Orthodontics, University of Pavia, Pavia, Italy

## Abstract

Objectives: The aim of this study was to assess the effect of blood contamination on the shear bond strength and failure site of three different orthodontic self-ligating brackets. Study Design: 240 bovine permanent mandibular incisors were randomly divided into 12 groups of 20 specimens each. Orthodontic self-ligating brackets were tested under four different enamel surface conditions: a) dry, b) blood contamination before priming, c) blood contamination after priming, d) blood contamination before and after priming. Brackets were bonded to the teeth and subsequently tested using a Instron universal testing machine. Shear bond strength values and adhesive failure rate were recorded. Statistical analysis was performed using ANOVA and Tukey tests (strength values), and Chi squared test (ARI Scores). Results: Non-contaminated enamel surfaces showed highest bond strengths for all self ligating brackets. Under blood-contamination shear bond strengths lowered for all brackets tested. Groups contaminated before and after primer application showed the lowest shear bond strength. Significant differences in debond locations were found among the groups under the various enamel surface conditions. Conclusions: Blood contamination of enamel during the bonding procedure lowers bond strength values of self ligating brackets, expecially when contamination occur in different times of the bonding procedure.

** Key words:**Disinclusion, self ligating brackets, blood, contamination, enamel, orthodontics, oral surgery.

## Introduction

Self-ligating brackets have been introducted for their advantages in orthodontic treatment: they are able to reduce unwanted friction (1), eliminate the requirement for elastomeric ligatures (2), insure more certain archwire engagement (3) and offer faster archwire removal and ligation (4). When orthodontists and surgeons collaborate in the exposure and orthodontic alignment of unerupted teeth, it could be useful to bond self-ligating bracket also onto ectopic teeth in order to reduce friction and speed orthodontic movement. In these cases it is difficult to maintain ideal working conditions and blood contamination during bonding can occur (5). Bonding of orthodontic brackets with composite resin adhesives requires a dry field of operation. Previous studies evaluating the effect of blood contamination on the bond strengths of ligh tcured composites showed a significant reduction in bond strength values (5-10). In fact when blood affects the bond, etched enamel becomes wet, most of the porosities become plugged, and resin penetration is impaired; this results in resin tags of insufficient numbers and lengths (11).

Nowadays in literature there are no published studies that compared shear bond strength of self ligating orthodontic bracket under bood contamination.

Accordingly, the aim of the present investigation was to measure and compare shear bond strength and adhesive remnant index score of three different self-ligating orthodontic brackets onto dry and blood-contaminated enamel. The null hypothesis of the study was that there is no significant difference in shear bond strength values and debond locations among the various groups.

## Material and Methods

Two hundred and forty freshly permanent extracted bovine mandibular incisors were collected from a local slaughterhouse and stored in a solution of 0.1% (wt/vol) thymol. The criteria for tooth selection included intact buccal enamel with no cracks caused by extraction and no caries. The teeth were cleansed of soft tissue and embedded in cold-curing, fast-setting acrylic (Leocryl, Leone, Sesto Fiorentino, Italy). Metal rings (15-mm diameter) were filled with the acrylic resin and allowed to cure, thus encasing each specimen while allowing the buccal surface of enamel to be exposed. Each tooth was oriented so that its labial surface was parallel to the shearing force. Teeth were randomly divided in 12 groups of 20 specimens.

Three different orthodontic self-ligating maxillary central incisor brackets were tested: Smart Clip (3M, Monrovia, Calif), Quick (Forestadent, Pforzheim, Germany), Damon 3MX (Ormco, Glendora, Calif). Brackets were tested under 4 different enamel surface conditions: (1) dry, (2) blood contamination before priming, (3) blood contamination after priming, (4) blood contamination before and after priming.

Before bonding, the labial surface of each incisor was cleaned for 10 seconds with a mixture of water and fluoride-free pumice in a rubber polishing cup with a low-speed handpiece. The enamel surface was rinsed with water to remove pumice or debris and then dried with an oil-free air stream.

Bonding procedures are described in ( Table 1). Teeth were etched with 37% phosphoric acid gel (3M Unitek, Monrovia, California) for 30 seconds, followed by thorough washing and drying. A thin layer of primer (Ortho Solo; Ormco, Glendora, California) was applied on the etched enamel, and then the brackets were bonded with a resin (Transbond XT, 3M Unitek, Monrovia, California) near the center of the facial surface of the teeth with sufficient pressure to express excess adhesive, which was removed from the margins of the bracket base with a scaler before polymerization. To achieve reproducible conditions, the teeth treated under moistened conditions were contaminated with fresh human blood from 1 female donor; the blood was applied with a brush onto the labial surfaces until they were totally contaminated.

**Table 1:**
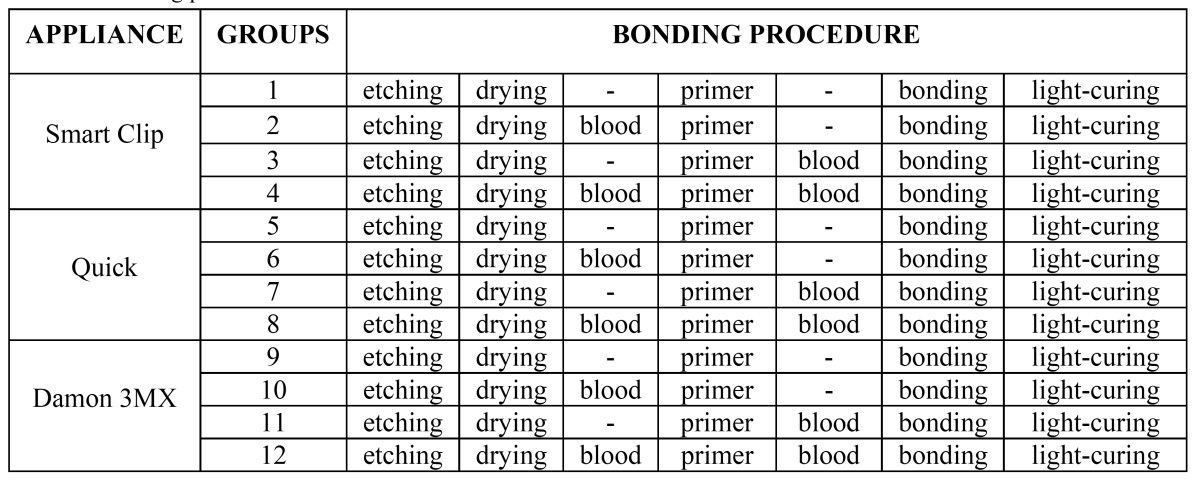
Bonding procedures for the different enamel surface conditions.

Brackets were then light-cured with a visible light-curing unit (Ortholux XT, 3M Unitek, Monrovia, California) for 10 seconds on the mesial side of the bracket and for 10 seconds on the distal side (total cure time 20 seconds). After bonding, all samples were stored in distilled water at room temperature for 24 hours and then tested in a shear mode with an universal testing machine (Model 3343, Instron, Canton, Massachussetts). Specimens were secured in the lower jaw of the machine so that the bonded bracket base was parallel to the shear force direction.

Specimens were stressed in an occlusogingival direction at a crosshead speed of 1 mm per minute, as in previous studies (12-14). The maximum load necessary to debond or initiate bracket fracture was recorded in newtons and then converted into MPa as a ratio of newtons to surface area of the bracket. After bond failure, the bracket bases and the enamel surfaces were examined under an optical microscope (Stereomicroscope SR, Zeiss, Oberkochen, Germany) at 10x magnification. The adhesive remnant index (ARI) was used to assess the amount of adhesive left on the enamel surface (15).

This scale ranges from 0 to 3. A score of 0 indicates no adhesive remaining on the tooth in the bonding area; 1 indicates less than half of the adhesive remaining on the tooth; 2 indicates more than half of the adhesive remaining on the tooth; and 3 indicates all adhesive remaining on the tooth. The ARI scores were used as a more complex method of defining bond failure site among the enamel, the adhesive, and the bracket base.

Statistical analysis was performed with Stata 11.0 software (Stata, College Station, Texas). Descriptive statistics, including the mean, standard deviation, median, and minimum and maximum values were calculated for all groups.

An analysis of variance (ANOVA) test was applied to determine whether significant differences in debond values existed among the groups. The Tukey test was used as post-hoc. The chi-square test was used to determine significant differences in the ARI scores among the different groups. Significance for all statistical tests was predetermined at P < 0.05.

## Results

**Figure 1:**
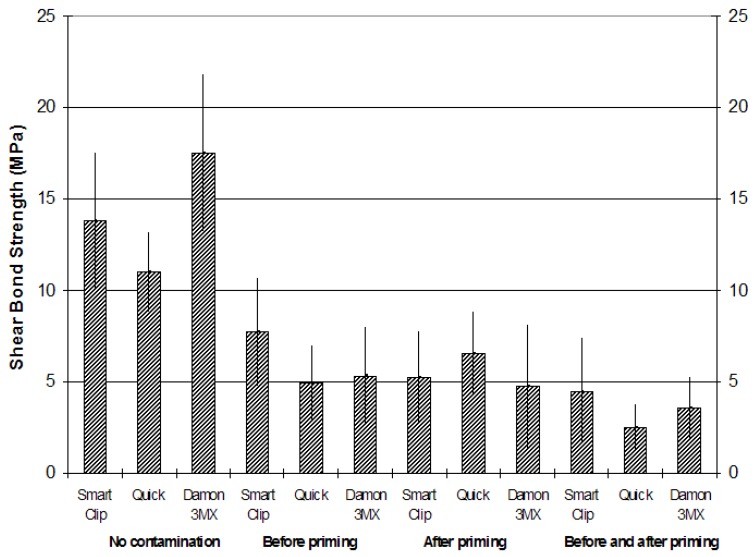
Mean shear bond strengths (MPa) of the three different brackets under the four different testing conditions (no contamination; blood contamination before priming; blood contamination after priming; blood contamination before and after priming).

Descriptive statistics for the shear bond strength (MPa) of the different brackets are illustrated in ( Table 2, Fig. 1). The normality of the data was calculated using the Kolmogorov-Smirnov test. The analysis of variance showed the presence of significant differences among the various groups (P < 0.05). Post-hoc test showed that brackets Smart Clip, Quick and Damon 3MX bonded onto dry enamel (groups 1, 2 and 3) presented the highest shear bond strength (P < 0.001). The three different brackets bonded under blood contamination before priming (groups 4, 5 and 6) or after priming (groups 7, 8 and 9) showed significantly lower shear bond strength (P < 0.01) and no significant differences among them (P > 0,05). Finally brackets bonded under blood contamination before and after priming (groups 10, 11 and 12) had lower bond strengths (P < 0.05), and showed no significant difference among them (P > 0.05).

**Table 2:**
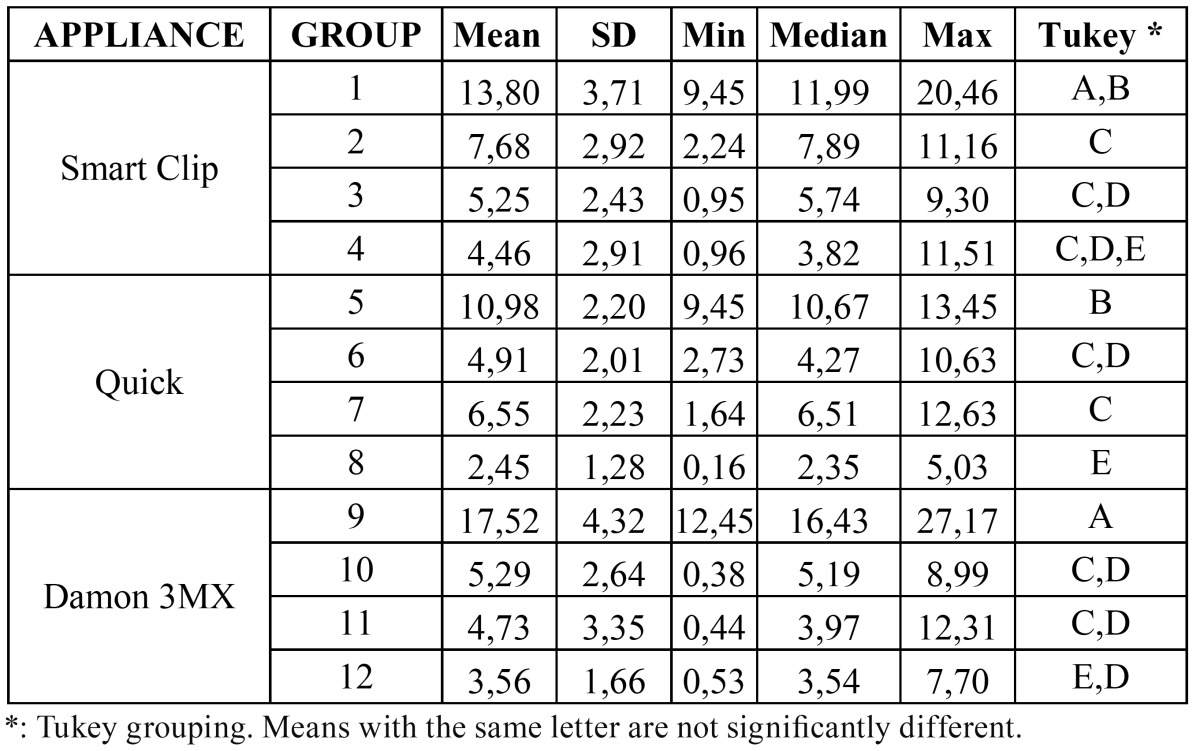
Descriptive statistics (in MPa) of shear bond strengths of the 12 groups tested (each group consisted of 20 specimens).

The results of ARI Scores are illustrated in ( Table 3). The chi-square test reported a higher frequency of ARI score of “2” for uncontaminated enamel groups (1,2 and 3) (P < 0.05) and exhibited no significant difference among them (P > 0.05). When blood contamination occurred before priming (Groups 4, 5 and 6) or after priming (groups 7, 8 and 9) a significantly higher frequency of ARI Score of “1” (P < 0.05) was reported. When blood contamination was applied before and after priming Smart Clip and Quick brackets (groups 10 and 11) showed significantly higher frequency of ARI Score of “1” (P < 0.05), whereas Damon 3MX brackets (group 12) showed significantly higher frequency of ARI Score of “0” (P < 0.01).

**Table 3:**
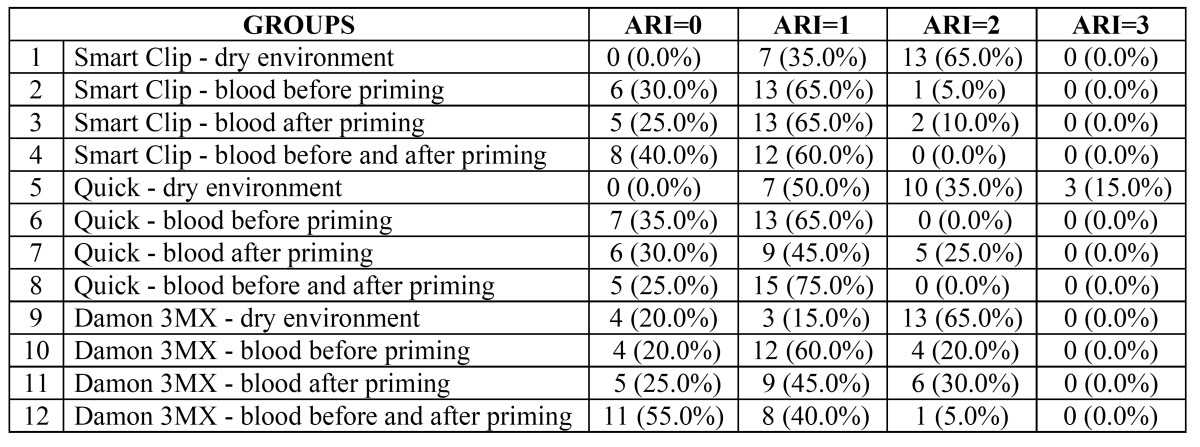
Frequency of distribution of adhesive remnant index scores (%).

## Discussion

The null hypothesis of the study has been rejected. In the present investigation self ligating brackets bonded onto dry enamel had significantly higher shear bond strength values than other groups. When contamination occurred before or after priming significantly lower shear bond strength values were recorded. Lowest shear strength values were obtained when blood contamination occurred both before and after priming. In literature self ligating brackets has been tested only onto dry enamel (16-18). To our knowledge there are no published studies that evaluated bond strength of self-ligating brackets onto blood contaminated enamel. Previous investigations that evaluated shear bond strength of blood contaminated conventional orthodontic brackets showed significant reduction of strength values compared to brackets bonded onto uncontaminated enamel (6-10,14,19).

Fluid contamination during bonding can lead to premature failure of the bond because when etched enamel becomes wet, the plugging of its porosities can affect resin penetration (20). This results in resin tags of insufficient number and length (11). In fact blood seems to be a physical barrier that impedes the mechanical retention of the adhesive to the etched tooth.

Enamel surface contamination can occur at different critical times of the bonding procedure: after the tooth surface has been etched, after the primer has been applied and after both procedures (14). In the present investigation no significant differences were found among groups bonded after blood contamination before priming (groups 4, 5 and 6) and among groups bonded after blood contamination after priming (groups 7, 8 and 9). When brackets were bonded under blood contaminated enamel before and after priming (groups 10, 11 and 12) unsignificant (groups 10 and 12) and significant (group 11) further strength values reduction has been reported. These results agree with those previously reported in other investigations using conventional brackets bonded with different adhesive systems on blood-moistened enamel surfaces (14,19).

In the present investigation permanent bovine lower incisors were used. Extracted human teeth are becoming difficult to obtain due to recent progress in conservative dental treatment. In order to find a substitute for human teeth the use of bovine lower incisors for dental experimental studies has been proposed for two main reasons. First, it is easier to obtain a sufficient number of sound bovine teeth than human teeth. Second, the bigger surface area of bovine lower incisors allows more accurate preparation. Bovine teeth, derived from animals of similar genetic lineage and dietary environment, might show higher homogeneity of mineral composition than different human teeth, which are collected from various donators with diverse dietary or fluoride supplementation (21). Only lower incisors were collected because they have a minimal curvature when compared to other bovine teeth, that allows a more accurate bracket placement (21).

Previous authors evaluated adhesive strength to bovine enamel and found no statistically significant difference between bovine and human teeth with any of the materials used, although the mean values were always slightly lower with bovine teeth (22). In fact, bovine enamel is similar to human enamel and generally the teeth of all mammals appears to be very similar on a histochemical and anatomic basis (23). Despite the differences, bovine enamel has been reported to be a reliable substitute for human enamel in bonding studies with similar or slightly lower strength values (22,24,25). Therefore, since bovine enamel has been reported to have similar physical properties, composition, and bond strengths than human enamel, it can be mimicking human teeth in general so it can be considered as a reliable substitute for human enamel in bonding studies (21-27).

A minimum bond strength of 6 to 8 MPa has been reported to be sufficient for most clinical orthodontic needs, because these values are considered able to withstand masticatory and orthodontic forces (28,29). In the present study, the bond strengths of the three different brackets tested bonded onto dry enamel surface were above these limits. When brackets were used on blood-contaminated enamel, the minimum requirement was achieved only in groups 2 (brackets Smart Clip under blood contamination before priming) and 7 (brackets Quick under blood contamination after priming). All the other contaminated groups showed mean shear strength values under the minimum required for orthodontic purposes. This is in agreement with previous investigations that evaluated shear bond strength of conventional orthodontic brackets bonded onto blood contaminated enamel (14,19,30).

Moreover in the present investigation ARI Scores has been recorded. Uncontaminated enamel groups reported a higher frequency of ARI score of “2”. Under blood contamination, brackets bonded with blood contamination before priming and groups with blood contamination after priming showed significantly higher frequency of ARI Score of “1”. When blood contamination was applied before and after priming Smart Clip and Quick brackets showed significantly higher frequency of ARI Score of “1”, whereas Damon 3MX brackets showed significantly higher frequency of ARI Score of “0”. This is in agreement with previous investigations that evaluated ARI Scores of orthodontic brackets bonded onto blood contaminated enamel (14,10,19) that all showed lower scores for blood contaminated groups (5,14,19,30).

The present study demonstrated that non-contaminated enamel surfaces showed highest bond strengths for all self-ligating brackets tested. Under blood-contamination all groups showed significantly lower shear bond strength values. Lowest shear bond strength values were recorded when blood application occurred before and after priming. Moreover non-contaminated groups showed higher frequency of ARI Score of “2”. For blood contaminated groups, orthodontic self-ligating brackets showed higher frequency of ARI Score of “1” and “0”.
